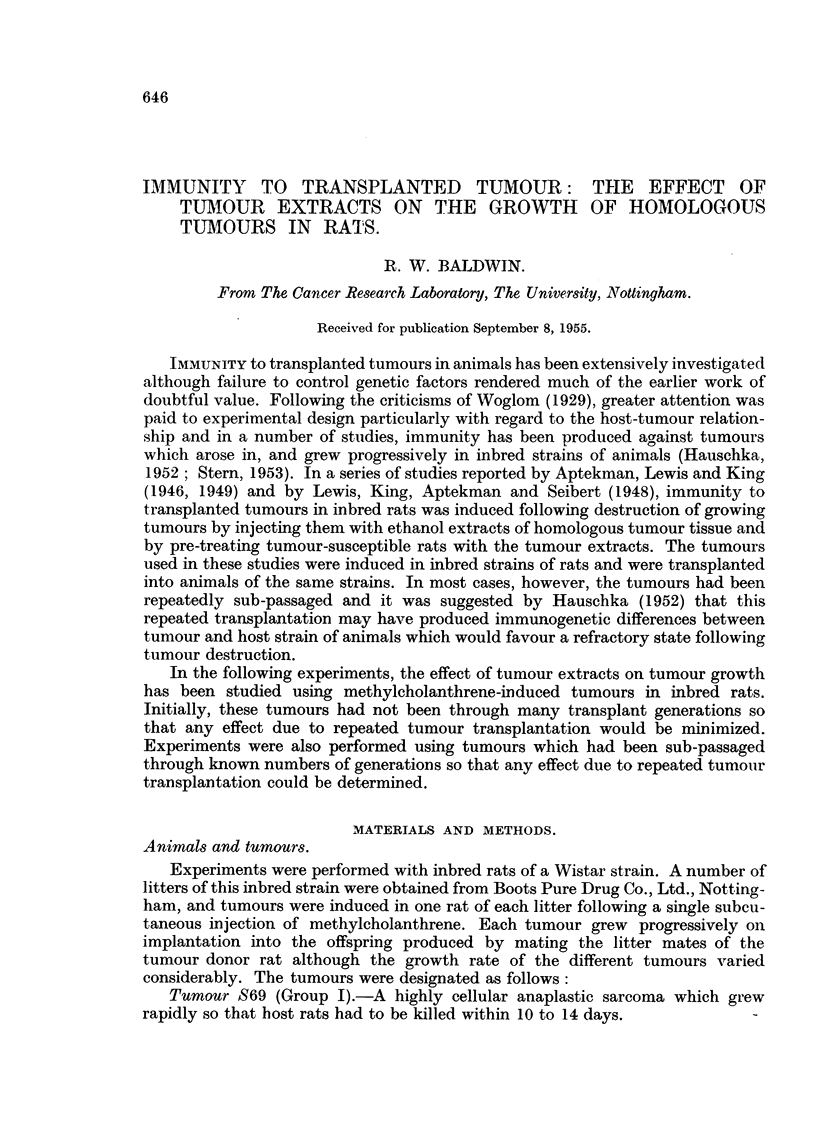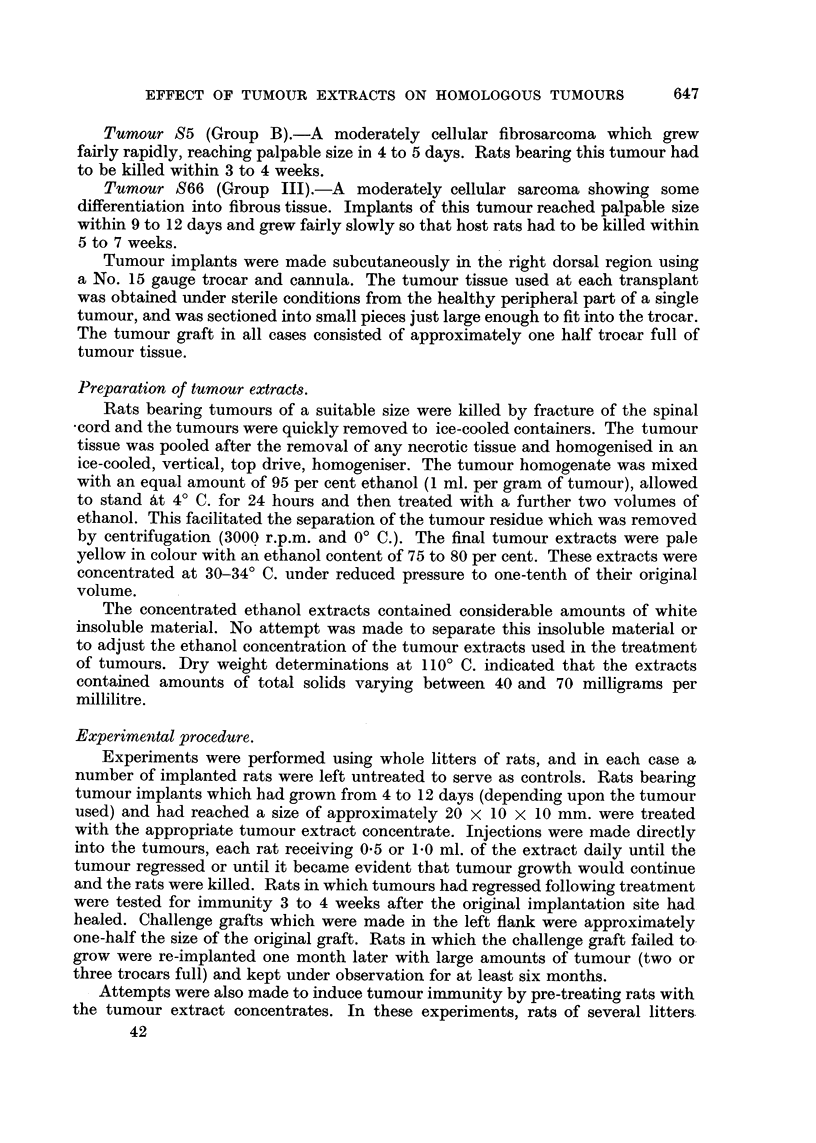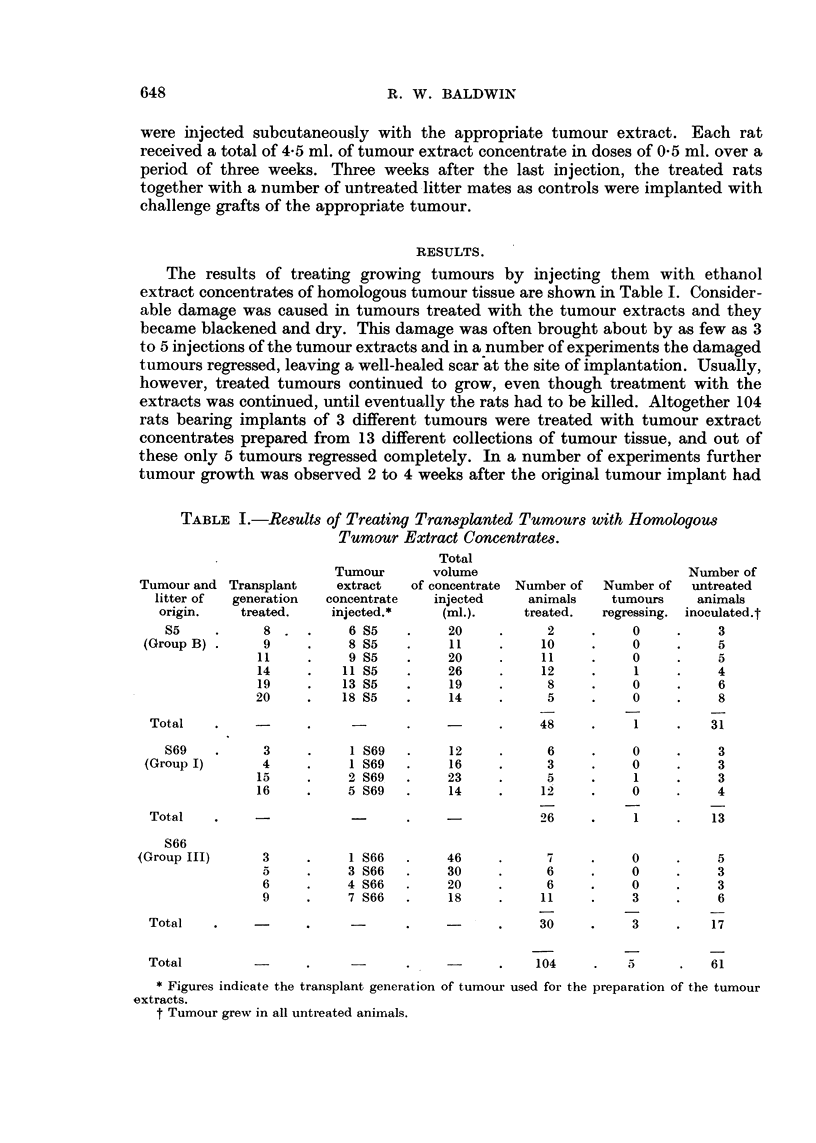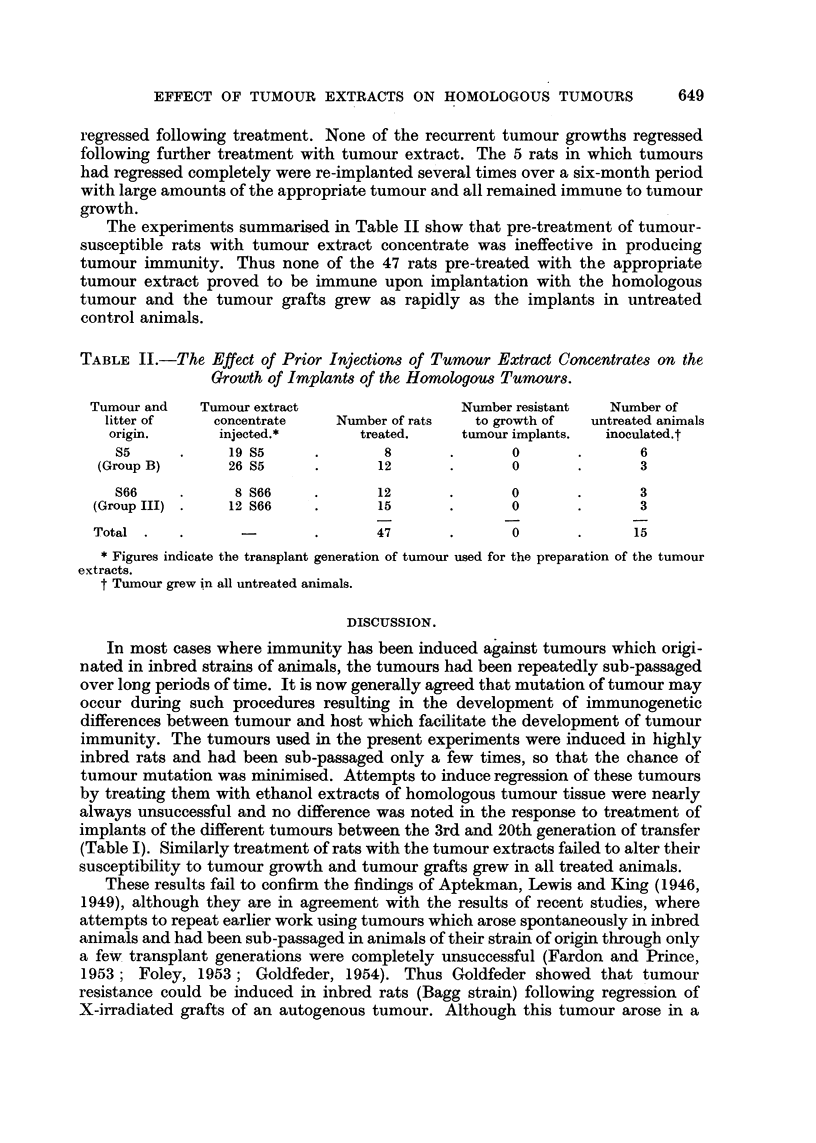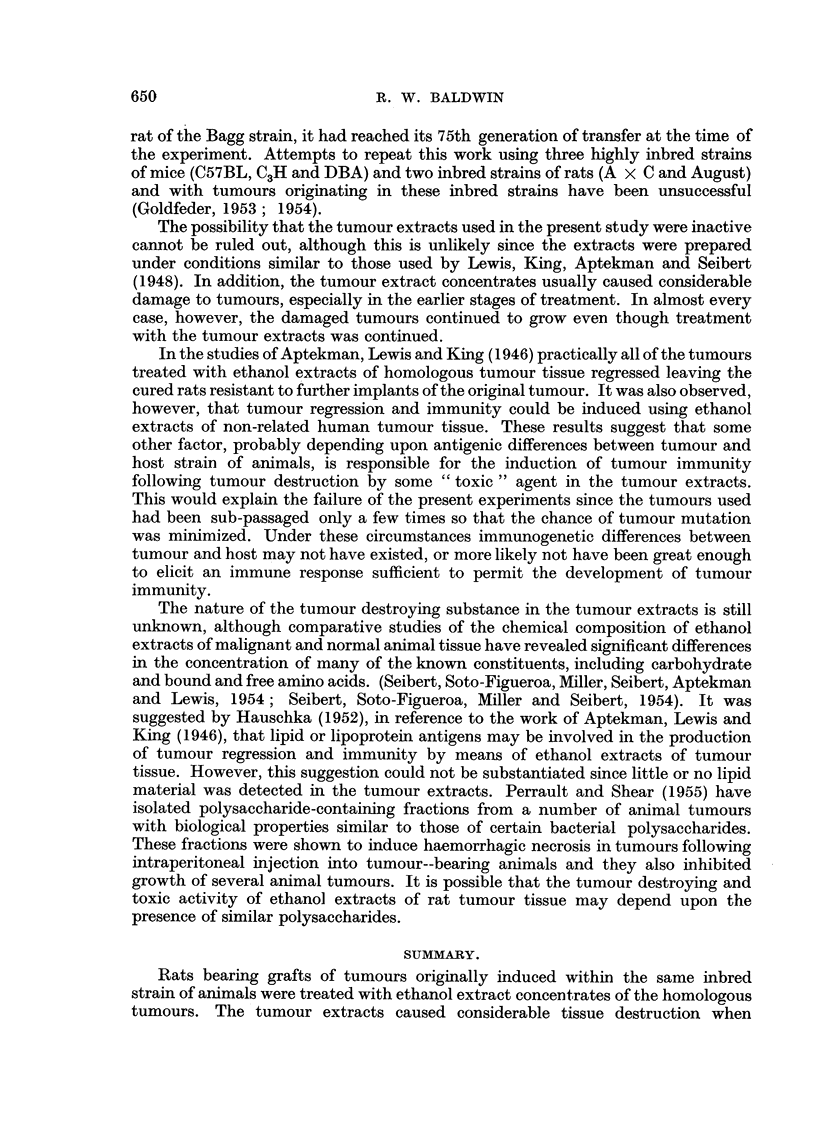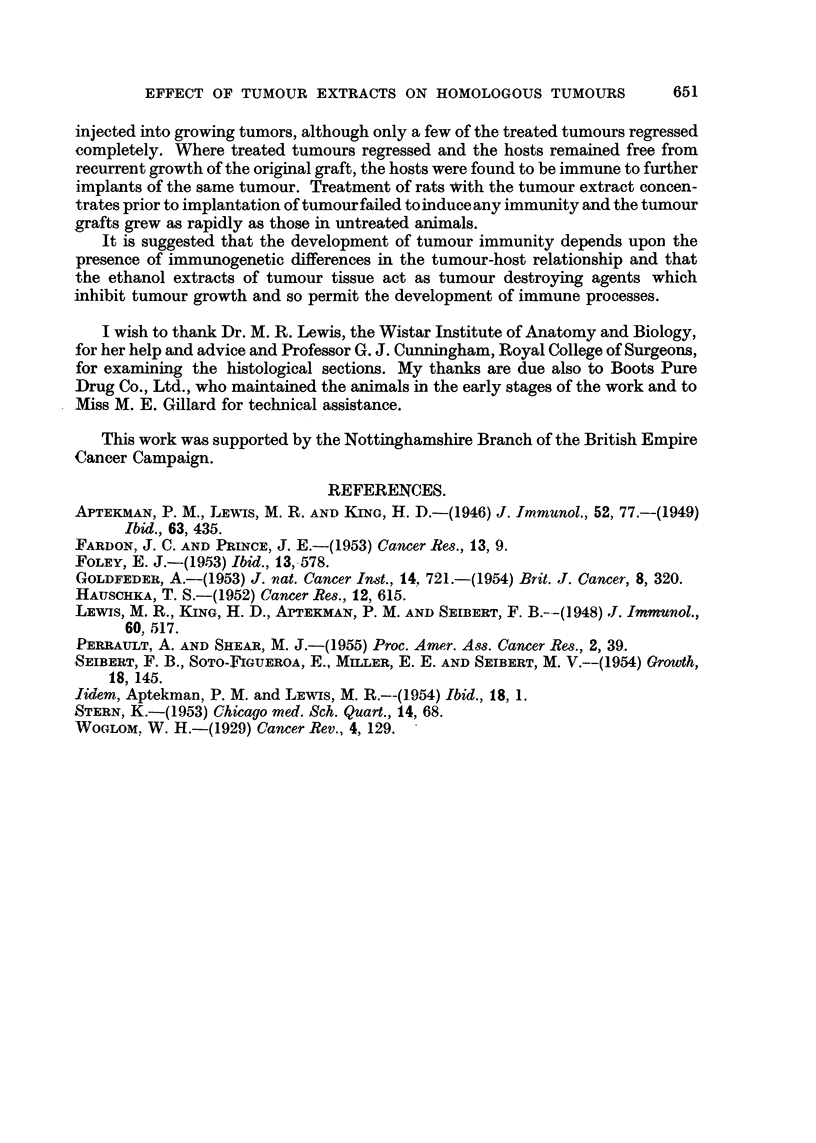# Immunity to Transplanted Tumour: The Effect of Tumour Extracts on the Growth of Homologous Tumours in Rats

**DOI:** 10.1038/bjc.1955.69

**Published:** 1955-12

**Authors:** R. W. Baldwin


### IMMUNITY TO TRANSPLANTED TUMOUR: THE EFFECT OF TUMOUR EXTRACTS ON THE GROWTH OF HOMOLOGOUS TUMOURS IN RATS.

R. W. BALDWIN.

*From The Cancer Research Laboratory, The University, Nottingham.*

Received for publication September 8, 1955.

IMMUNITY to transplanted tumours in animals has been extensively investigated although failure to control genetic factors rendered much of the earlier work of doubtful value. Following the criticisms of Woglom (1929), greater attention was paid to experimental design particularly with regard to the host-tumour relationship and in a number of studies, immunity has been produced against tumours which arose in, and grew progressively in inbred strains of animals (Hauschka, 1952; Stern, 1953). In a series of studies reported by Aptekman, Lewis and King (1946, 1949) and by Lewis, King, Aptekman and Seibert (1948), immunity to transplanted tumours in inbred rats was induced following destruction of growing tumours by injecting them with ethanol extracts of homologous tumour tissue and by pre-treating tumour-susceptible rats with the tumour extracts. The tumours used in these studies were induced in inbred strains of rats and were transplanted into animals of the same strains. In most cases, however, the tumours had been repeatedly sub-passaged and it was suggested by Hauschka (1952) that this repeated transplantation may have produced immunogenetic differences between tumour and host strain of animals which would favour a refractory state following tumour destruction.

In the following experiments, the effect of tumour extracts on tumour growth has been studied using methylcholanthrene-induced tumours in inbred rats. Initially, these tumours had not been through many transplant generations so that any effect due to repeated tumour transplantation would be minimized. Experiments were also performed using tumours which had been sub-passaged through known numbers of generations so that any effect due to repeated tumour transplantation could be determined.

### MATERIALS AND METHODS.

### *Animals and tumours.*

Experiments were performed with inbred rats of a Wistar strain. A number of litters of this inbred strain were obtained from Boots Pure Drug Co., Ltd., Nottingham, and tumours were induced in one rat of each litter following a single subcutaneous injection of methylcholanthrene. Each tumour grew progressively on implantation into the offspring produced by mating the litter mates of the tumour donor rat although the growth rate of the different tumours varied considerably. The tumours were designated as follows:

*Tumour S69* (Group I).—A highly cellular anaplastic sarcoma which grew rapidly so that host rats had to be killed within 10 to 14 days.

*Tumour S*5 (Group B).—A moderately cellular fibrosarcoma which grew fairly rapidly, reaching palpable size in 4 to 5 days. Rats bearing this tumour had to be killed within 3 to 4 weeks.

*Tumour S*66 (Group III).—A moderately cellular sarcoma showing some differentiation into fibrous tissue. Implants of this tumour reached palpable size within 9 to 12 days and grew fairly slowly so that host rats had to be killed within 5 to 7 weeks.

Tumour implants were made subcutaneously in the right dorsal region using a No. 15 gauge trocar and cannula. The tumour tissue used at each transplant was obtained under sterile conditions from the healthy peripheral part of a single tumour, and was sectioned into small pieces just large enough to fit into the trocar. The tumour graft in all cases consisted of approximately one half trocar full of tumour tissue.

*Preparation of tumour extracts.*

Rats bearing tumours of a suitable size were killed by fracture of the spinal cord and the tumours were quickly removed to ice-cooled containers. The tumour tissue was pooled after the removal of any necrotic tissue and homogenised in an ice-cooled, vertical, top drive, homogeniser. The tumour homogenate was mixed with an equal amount of 95 per cent ethanol (1 ml. per gram of tumour), allowed to stand at 4° C. for 24 hours and then treated with a further two volumes of ethanol. This facilitated the separation of the tumour residue which was removed by centrifugation (3000 r.p.m. and 0° C.). The final tumour extracts were pale yellow in colour with an ethanol content of 75 to 80 per cent. These extracts were concentrated at 30–34° C. under reduced pressure to one-tenth of their original volume.

The concentrated ethanol extracts contained considerable amounts of white insoluble material. No attempt was made to separate this insoluble material or to adjust the ethanol concentration of the tumour extracts used in the treatment of tumours. Dry weight determinations at 110° C. indicated that the extracts contained amounts of total solids varying between 40 and 70 milligrams per millilitre.

*Experimental procedure.*

Experiments were performed using whole litters of rats, and in each case a number of implanted rats were left untreated to serve as controls. Rats bearing tumour implants which had grown from 4 to 12 days (depending upon the tumour used) and had reached a size of approximately 20 × 10 × 10 mm. were treated with the appropriate tumour extract concentrate. Injections were made directly into the tumours, each rat receiving 0·5 or 1·0 ml. of the extract daily until the tumour regressed or until it became evident that tumour growth would continue and the rats were killed. Rats in which tumours had regressed following treatment were tested for immunity 3 to 4 weeks after the original implantation site had healed. Challenge grafts which were made in the left flank were approximately one-half the size of the original graft. Rats in which the challenge graft failed to grow were re-implanted one month later with large amounts of tumour (two or three trocars full) and kept under observation for at least six months.

Attempts were also made to induce tumour immunity by pre-treating rats with the tumour extract concentrates. In these experiments, rats of several litters were injected subcutaneously with the appropriate tumour extract. Each rat received a total of 4·5 ml. of tumour extract concentrate in doses of 0·5 ml. over a period of three weeks. Three weeks after the last injection, the treated rats together with a number of untreated litter mates as controls were implanted with challenge grafts of the appropriate tumour.

### RESULTS.

The results of treating growing tumours by injecting them with ethanol extract concentrates of homologous tumour tissue are shown in Table I. Considerable damage was caused in tumours treated with the tumour extracts and they became blackened and dry. This damage was often brought about by as few as 3 to 5 injections of the tumour extracts and in a number of experiments the damaged tumours regressed, leaving a well-healed scar at the site of implantation. Usually, however, treated tumours continued to grow, even though treatment with the extracts was continued, until eventually the rats had to be killed. Altogether 104 rats bearing implants of 3 different tumours were treated with tumour extract concentrates prepared from 13 different collections of tumour tissue, and out of these only 5 tumours regressed completely. In a number of experiments further tumour growth was observed 2 to 4 weeks after the original tumour implant had

TABLE I.—*Results of Treating Transplanted Tumours with Homologous Tumour Extract Concentrates.*

| Tumour and litter of origin. | Transplant generation treated. | Tumour extract concentrate injected.* | Total volume of concentrate injected (ml.). | Number of animals treated. | Number of tumours regressing. | Number of untreated animals inoculated.† |
|---|---|---|---|---|---|---|
| S5 | 8 | 6 S5 | 20 | 2 | 0 | 3 |
| (Group B) | 9 | 8 S5 | 11 | 10 | 0 | 5 |
| | 11 | 9 S5 | 20 | 11 | 0 | 5 |
| | 14 | 11 S5 | 26 | 12 | 1 | 4 |
| | 19 | 13 S5 | 19 | 8 | 0 | 6 |
| | 20 | 18 S5 | 14 | 5 | 0 | 8 |
| Total | — | — | — | 48 | 1 | 31 |
| S69 | 3 | 1 S69 | 12 | 6 | 0 | 3 |
| (Group I) | 4 | 1 S69 | 16 | 3 | 0 | 3 |
| | 15 | 2 S69 | 23 | 5 | 1 | 3 |
| | 16 | 5 S69 | 14 | 12 | 0 | 4 |
| Total | — | — | — | 26 | 1 | 13 |
| S66 | | | | | | |
| (Group III) | 3 | 1 S66 | 46 | 7 | 0 | 5 |
| | 5 | 3 S66 | 30 | 6 | 0 | 3 |
| | 6 | 4 S66 | 20 | 6 | 0 | 3 |
| | 9 | 7 S66 | 18 | 11 | 3 | 6 |
| Total | — | — | — | 30 | 3 | 17 |
| Total | — | — | — | 104 | 5 | 61 |

* Figures indicate the transplant generation of tumour used for the preparation of the tumour extracts.

† Tumour grew in all untreated animals.

regressed following treatment. None of the recurrent tumour growths regressed following further treatment with tumour extract. The 5 rats in which tumours had regressed completely were re-implanted several times over a six-month period with large amounts of the appropriate tumour and all remained immune to tumour growth.

The experiments summarised in Table II show that pre-treatment of tumour-susceptible rats with tumour extract concentrate was ineffective in producing tumour immunity. Thus none of the 47 rats pre-treated with the appropriate tumour extract proved to be immune upon implantation with the homologous tumour and the tumour grafts grew as rapidly as the implants in untreated control animals.

TABLE II.—*The Effect of Prior Injections of Tumour Extract Concentrates on the Growth of Implants of the Homologous Tumours.*

| Tumour and litter of origin. | Tumour extract concentrate injected.* | Number of rats treated. | Number resistant to growth of tumour implants. | Number of untreated animals inoculated.† |
|---|---|---|---|---|
| S5 | 19 S5 | 8 | 0 | 6 |
| (Group B) | 26 S5 | 12 | 0 | 3 |
| S66 | 8 S66 | 12 | 0 | 3 |
| (Group III) | 12 S66 | 15 | 0 | 3 |
| Total | — | 47 | 0 | 15 |

* Figures indicate the transplant generation of tumour used for the preparation of the tumour extracts.

† Tumour grew in all untreated animals.

### DISCUSSION.

In most cases where immunity has been induced against tumours which originated in inbred strains of animals, the tumours had been repeatedly sub-passaged over long periods of time. It is now generally agreed that mutation of tumour may occur during such procedures resulting in the development of immunogenetic differences between tumour and host which facilitate the development of tumour immunity. The tumours used in the present experiments were induced in highly inbred rats and had been sub-passaged only a few times, so that the chance of tumour mutation was minimised. Attempts to induce regression of these tumours by treating them with ethanol extracts of homologous tumour tissue were nearly always unsuccessful and no difference was noted in the response to treatment of implants of the different tumours between the 3rd and 20th generation of transfer (Table I). Similarly treatment of rats with the tumour extracts failed to alter their susceptibility to tumour growth and tumour grafts grew in all treated animals.

These results fail to confirm the findings of Aptekman, Lewis and King (1946, 1949), although they are in agreement with the results of recent studies, where attempts to repeat earlier work using tumours which arose spontaneously in inbred animals and had been sub-passaged in animals of their strain of origin through only a few transplant generations were completely unsuccessful (Fardon and Prince, 1953; Foley, 1953; Goldfeder, 1954). Thus Goldfeder showed that tumour resistance could be induced in inbred rats (Bagg strain) following regression of X-irradiated grafts of an autogenous tumour. Although this tumour arose in a rat of the Bagg strain, it had reached its 75th generation of transfer at the time of the experiment. Attempts to repeat this work using three highly inbred strains of mice (C57BL, $C_3H$ and DBA) and two inbred strains of rats (A $\times$ C and August) and with tumours originating in these inbred strains have been unsuccessful (Goldfeder, 1953 ; 1954).

The possibility that the tumour extracts used in the present study were inactive cannot be ruled out, although this is unlikely since the extracts were prepared under conditions similar to those used by Lewis, King, Aptekman and Seibert (1948). In addition, the tumour extract concentrates usually caused considerable damage to tumours, especially in the earlier stages of treatment. In almost every case, however, the damaged tumours continued to grow even though treatment with the tumour extracts was continued.

In the studies of Aptekman, Lewis and King (1946) practically all of the tumours treated with ethanol extracts of homologous tumour tissue regressed leaving the cured rats resistant to further implants of the original tumour. It was also observed, however, that tumour regression and immunity could be induced using ethanol extracts of non-related human tumour tissue. These results suggest that some other factor, probably depending upon antigenic differences between tumour and host strain of animals, is responsible for the induction of tumour immunity following tumour destruction by some " toxic " agent in the tumour extracts. This would explain the failure of the present experiments since the tumours used had been sub-passaged only a few times so that the chance of tumour mutation was minimized. Under these circumstances immunogenetic differences between tumour and host may not have existed, or more likely not have been great enough to elicit an immune response sufficient to permit the development of tumour immunity.

The nature of the tumour destroying substance in the tumour extracts is still unknown, although comparative studies of the chemical composition of ethanol extracts of malignant and normal animal tissue have revealed significant differences in the concentration of many of the known constituents, including carbohydrate and bound and free amino acids. (Seibert, Soto-Figueroa, Miller, Seibert, Aptekman and Lewis, 1954 ; Seibert, Soto-Figueroa, Miller and Seibert, 1954). It was suggested by Hauschka (1952), in reference to the work of Aptekman, Lewis and King (1946), that lipid or lipoprotein antigens may be involved in the production of tumour regression and immunity by means of ethanol extracts of tumour tissue. However, this suggestion could not be substantiated since little or no lipid material was detected in the tumour extracts. Perrault and Shear (1955) have isolated polysaccharide-containing fractions from a number of animal tumours with biological properties similar to those of certain bacterial polysaccharides. These fractions were shown to induce haemorrhagic necrosis in tumours following intraperitoneal injection into tumour--bearing animals and they also inhibited growth of several animal tumours. It is possible that the tumour destroying and toxic activity of ethanol extracts of rat tumour tissue may depend upon the presence of similar polysaccharides.

### SUMMARY.

Rats bearing grafts of tumours originally induced within the same inbred strain of animals were treated with ethanol extract concentrates of the homologous tumours. The tumour extracts caused considerable tissue destruction when injected into growing tumors, although only a few of the treated tumours regressed completely. Where treated tumours regressed and the hosts remained free from recurrent growth of the original graft, the hosts were found to be immune to further implants of the same tumour. Treatment of rats with the tumour extract concentrates prior to implantation of tumour failed to induce any immunity and the tumour grafts grew as rapidly as those in untreated animals.

It is suggested that the development of tumour immunity depends upon the presence of immunogenetic differences in the tumour-host relationship and that the ethanol extracts of tumour tissue act as tumour destroying agents which inhibit tumour growth and so permit the development of immune processes.

I wish to thank Dr. M. R. Lewis, the Wistar Institute of Anatomy and Biology, for her help and advice and Professor G. J. Cunningham, Royal College of Surgeons, for examining the histological sections. My thanks are due also to Boots Pure Drug Co., Ltd., who maintained the animals in the early stages of the work and to Miss M. E. Gillard for technical assistance.

This work was supported by the Nottinghamshire Branch of the British Empire Cancer Campaign.

### REFERENCES.

APTEKMAN, P. M., LEWIS, M. R. AND KING, H. D.—(1946) *J. Immunol.*, **52**, 77.—(1949) *Ibid.*, **63**, 435.
FARDON, J. C. AND PRINCE, J. E.—(1953) *Cancer Res.*, **13**, 9.
FOLEY, E. J.—(1953) *Ibid.*, **13**, 578.
GOLDFEDER, A.—(1953) *J. nat. Cancer Inst.*, **14**, 721.—(1954) *Brit. J. Cancer*, **8**, 320.
HAUSCHKA, T. S.—(1952) *Cancer Res.*, **12**, 615.
LEWIS, M. R., KING, H. D., APTEKMAN, P. M. AND SEIBERT, F. B.—(1948) *J. Immunol.*, **60**, 517.
PERRAULT, A. AND SHEAR, M. J.—(1955) *Proc. Amer. Ass. Cancer Res.*, **2**, 39.
SEIBERT, F. B., SOTO-FIGUEROA, E., MILLER, E. E. AND SEIBERT, M. V.—(1954) *Growth*, **18**, 145.
*Iidem,* Aptekman, P. M. and LEWIS, M. R.—(1954) *Ibid.*, **18**, 1.
STERN, K.—(1953) *Chicago med. Sch. Quart.*, **14**, 68.
WOGLOM, W. H.—(1929) *Cancer Rev.*, **4**, 129.